# A multi-level perspective on perceived unmet needs for home support in home-dwelling older adults in the Swiss context: a secondary data analysis of a population study

**DOI:** 10.1186/s12877-022-03479-5

**Published:** 2022-11-03

**Authors:** Maria Jose Mendieta, Sabina M De Geest, Geert Goderis, Olivia Yip, Mieke Deschodt, Suzanne Dhaini, the INSPIRE Consortium

**Affiliations:** 1 Department of Public Health, Institute of Nursing Science, University of Basel, Basel, Switzerland; 2 Department of Public Health and Primary Care, Academic Centre for Nursing and Midwifery, KU Leuven, Leuven, Belgium; 3 Department of Public Health and Primary Care, Academic Center of General Practice, KU Leuven, Leuven Belgium; 4 Department of Public Health and Primary Care, Gerontology and Geriatrics, KU Leuven, Leuven Belgium; 5 Competence Center of Nursing, University Hospitals Leuven, Leuven, Belgium; 6 Medizinische Fakultät, Department of Public Health (DPH), Universität Basel, Bernoullistrasse 28, 4056 Basel, Switzerland

**Keywords:** Home-dwelling older adults, Unmet needs, Home support, Ecological model, Socio-economical inequality

## Abstract

**Background:**

Unmet needs for home support occur when any support services perceived by older people as needed are not being received. Not meeting these needs can negatively impact older adults’ quality of life, and increase health care utilization, hospitalizations, institutionalizations, or death. To date there is no consensus in how to define and assess these unmet needs. In parallel, previous research of factors associated with unmet needs for home support has mostly focused on factors at the micro level. Thus, this paper aims to identify the prevalence of unmet needs for home support among a home-dwelling older population and the factors at the macro, meso and micro levels contributing to them.

**Methods:**

Using an ecological approach we identified multi-level factors associated with the presence of unmet needs for home support among the home-dwelling older population (aged 75+) in Switzerland. This is a secondary cross-sectional analysis of the INSPIRE Population Survey of home-dwelling older adults (n = 8,508) living in Basel-Landschaft in Switzerland, conducted as part of the TRANS-SENIOR Project. Prevalence of perceived unmet needs for home support was self-reported, using a dichotomized question. Multiple logistic regression analyses were performed to investigate the associations of factors at each level with unmet needs for home support.

**Results:**

4.3% of participants reported unmet needs for home support, with a median age of 81 years. 45.1% had private health insurance and 6.3% needed additional government support. Being a recipient of other type of government support (OR = 1.65; 95% CI = 1.17–2.29) (macro-); the use of transportation services (OR = 1.74; 95% CI = 1.15–2.57) (meso-); and feeling depressed (OR = 1.40; 95% CI = 1.06–1.85) or abandoned (OR = 2.60; 95% CI = 1.96–3.43) (micro-) increased odds of having perceived unmet needs for home support. Having a private health insurance (macro-) (OR = 0.63; 95% CI = 0.49–0.80), speaking Swiss-German (OR = 0.44; 95% CI = 0.24–0.88) or German (OR = 0.47; 95% CI = 0.24–0.98), having a high level of education [primary (OR = 0.48; 95% CI = 0.24–1.02); secondary (OR = 0.49; 95% CI = 0.25–1.03); tertiary (OR = 0.38; 95% CI = 0.19–0.82); other (OR = 0.31 (0.12–0.75)], having a high score of self-perceived health status [score ≥ 76 (OR = 0.42; 95% CI = 0.20–0.96)] and having informal care (OR = 0.57; 95% CI = 0.45–0.73), among others (micro-) were associated with decreased odds of having perceived unmet needs for home support.

**Conclusion:**

Our study findings highlight the role of socio-economical inequality in the perception of unmet needs for home support in home-dwelling older adults. In order to address unmet needs in home-dwelling older adults, healthcare leaders and policy makers should focus on strategies to reduce socio-economic inequalities at the different levels in this population.

**Supplementary Information:**

The online version contains supplementary material available at 10.1186/s12877-022-03479-5.

## Background

Unmet needs in home-dwelling older adults occur when services are not received or are insufficient to meet health care or social care needs [1–4]. Services needed can include health care support such as promotion, prevention, treatment, or rehabilitation services as well as home support such as support services to perform activities of daily living (ADL), instrumental activities of daily living (IADL), transportation, finances, or basic maintenance [1, 5]. Failure to meet the needs of health care or home support services can result in a lower quality of life of the older adult, increased health care utilization (e.g., physician visits) and an increased risk of hospitalizations, institutionalizations or death [2, 5–7].

The assessment of unmet needs for home support can be conducted in different ways, either a professional identifies them using standardized questionnaires, or it is self-reported by the older person, or a proxy respondent [1, 3]. But most of the time, this assessment focuses only on a specific type of support needed [3, 6]. For instance, in a study in the UK on unmet needs for home support, 55% of adults aged 65 and over reported unmet needs to perform ADL, and 24% to perform IADLs [3]. In the US, 11% of older adults of 65 years and more reported unmet needs for transportation [6]. Despite the attempt to define and measure unmet needs for home support, there is no consensus on the concept and how to assess it in older adults [4]. The most used definition involves asking the older person about their difficulties to perform ADLs or IADLs. When those difficulties are present, and no- support is provided, this is defined as an unmet need. However, this definition does not incorporate the older person’s own perspective of whether their needs are met, instead assuming that once help is provided the need is met [4, 8]. Another disadvantage is that it is a task-oriented definition (e.g. support to get dressed), leaving out important areas such as social interaction and companionship [9]. An alternative approach involves asking the person directly whether they perceive that their needs are met or not. A consideration in this definition is that some older adults will suggest that their needs are unmet even when they are receiving help, and this could reflect that the services received are not enough or not fully suitable [4]. Therefore, in the current paper we refer to unmet needs for home support as any support for everyday activities that older people perceive as necessary but are not being received.

In order to better understand the factors that influence the older population’s health and capacity to cope with everyday activities, it is crucial to consider the complex environmental context in which the home-dwelling older person is embedded. Hence, employing an ecological model can provide insight into how the older person’s perceived problems and needs (micro level) interact with the overlapping layers of environmental contexts (the macro and meso levels), surrounding the older adult [10]. To date, most studies on unmet needs for home support have focused on factors related directly to the individual (micro level). Being older, female, living alone, having a lower socioeconomical status, residing in a rural area, having a poor self-rated life satisfaction and self-rated health have been associated with having unmet needs for home support [2, 11, 12]. There have been few efforts to explore how services in the community (meso level) and the system at large, e.g. national policies (macro level) are associated with meeting needs for support at home in older adults. Blake and colleagues reported that lack of transportation, geographical distances, higher costs of services, and unawareness of the services available or how to gain access to them, contribute to unmet needs in this population [9]. Similarly, the lack of budget, legislative support, or strict requirements to access support services have been denoted as factors of the system that contribute to unmet needs [6]. To the best of our knowledge, none of the previous studies have attempted to investigate the impact of macro, meso, and micro factors on unmet needs for home support using this ecological perspective.

To allow all people to retire with a considerable financial autonomy to cover their basic needs and remain active in the community, Switzerland instituted an old-age insurance system in 1947 [13]. Additionally, the country introduced a reform in the health system in 1994 delegating, for example, the provision of long-term care (nursing homes and home care services) to Cantons [14]. While evidence on the prevalence of unmet needs for support among older adults in Switzerland is lacking, data of the Federal Statistical Office demonstrated that 40.9% of older adults report some limitations in their daily activities [15]. This is in contrast with the 2.7% of older men and 4.5% of older women that reported the use of home support services [15]. Despite the insurance system for old age, and the high decentralization for the provision of services, the fact that only a small proportion of Swiss older adults are using home support services justifies the need to gain a better understanding of factors at both macro and meso levels, associated with unmet needs for home support. This information could help develop targeted interventions to improve support needs coverage. Therefore, the aim of this paper is to identify the prevalence of unmet needs for home support and the factors at the macro, meso and micro levels which contribute to them among a home-dwelling older population.

### Theoretical background & study framework

**Fig. 1:**
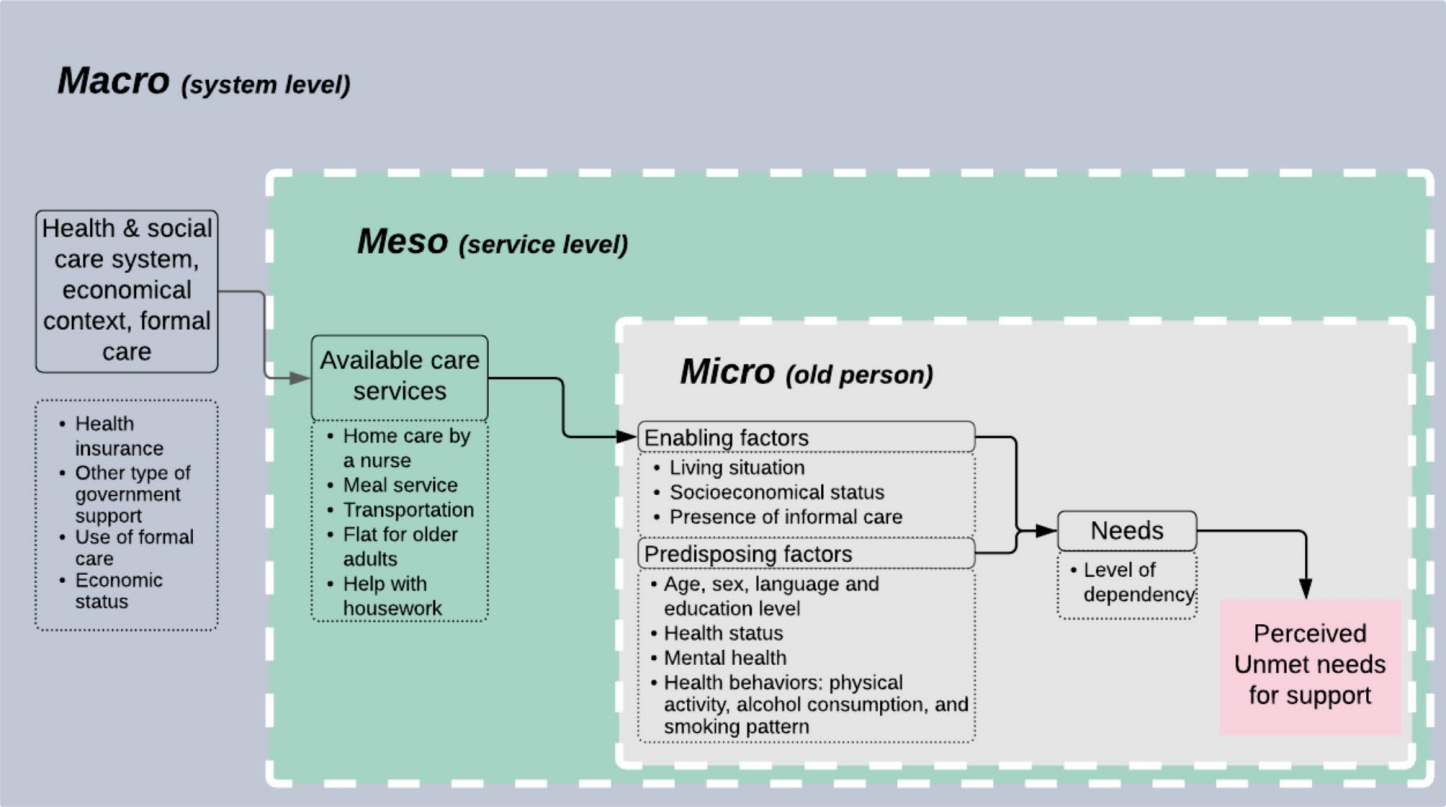
Study Framework

As there is currently not a well-defined framework to assess unmet needs for home support in home-dwelling older adults, we developed a framework integrating the main concepts of two theoretical models: the Andersen Behavioral Model of health services use and the Integrated Care for Older People (ICOPE) implementation framework [16, 17]. The Andersen behavioral model has been widely used to describe factors associated with older adults’ unmet needs for health care [6, 11, 12, 16, 18]. It highlights the impact of predisposing factors of an individual on their enabling factors to use existing services, without considering factors at other levels (i.e. macro and meso levels) [16]. On the other hand, the ICOPE framework, developed by the WHO, is grounded in the ecological model and constitutes a guide to address the needs of older adults with multimorbidity at three different levels (macro, meso and micro) [17]. Yet, it lacks the operationalization of the concepts described by Anderson. Consequently, using the ecological perspective of the ICOPE framework and guided by the concepts defined by Andersen, our study framework describes the interaction of factors at each level (macro, meso and micro level), and their impact on the development of the older adult’s needs. The macro level includes factors related to health and social care system, the economical context and the provision of formal care support; the meso level includes factors related to the available care services in the community and the micro level includes the enabling and predisposing factors of the older person (Fig. 1).

## Methods

### Design and sample

This is an exploratory secondary analysis using data from the INSPIRE (ImplemeNtation of a community-baSed care Program for home dwelling senIoR citizEns) Population Survey of older adults of the Canton of Basel-Landschaft (BL) (Bevölkerungsbefragung Basel-Landschaft in German). INSPIRE is a multi-phase implementation science project designed to develop, implement, and evaluate an integrated care model for home-dwelling older adults in the Canton BL in Switzerland [19]. The INSPIRE Population Survey was conducted between March and August 2019 as part of the contextual analysis of the INSPIRE project, to determine the current state, wishes, and needs of older adults with regard to their health, social support, and life situation.

### Sample

Following a population-based approach, the INSPIRE paper questionnaire, in German language, was mailed to all home-dwelling older adults who were 75 or older (29,045 people), living in urban and rural areas of the Canton BL in 2019. 8,846 questionnaires were received back (total response rate of 30.7%) [19, 20]. In the current study, our sample included home-dwelling older adults aged 75 years old and over, living in the Canton BL. Hence, the total sample included in the analysis was 8,543 older adults (see additional file 2).

### Setting

The old-age insurance system of Switzerland relies on three pillars: first pillar, state provision (funded through old-age pension Insurance - OASI); second pillar, occupational pension insurance (only for salaried workers); and third pillar, private provision. When this system is not enough to cover basic living costs, additional benefits are granted (i.e. supplementary benefits or helplessness compensation) [21]. The organization of long-term care (institutional care and home care services) is a responsibility of Cantons, but it is frequently delegated to municipalities (or communes). Institutional care is provided by nursing homes, while home care services are provided by public or private social care organizations [14].

### Variables and measurements

The methodology for the development of the questionnaire for the INSPIRE Population Survey has been published elsewhere [19, 20]. The selection of variables used in the current paper are below. Further details are summarized in **additional file 1.**

### Outcome variable

Unmet needs were identified when a person responded that the support they received in everyday life did not meet their needs. This question did not include a differentiation of the type of needs (social or health). A single question with a dichotomized answer (yes/no) was used for this purpose, where a positive answer corresponded to the presence of unmet needs.

#### Predictor variables at the macro level

Health insurance type was assessed by asking the older person the type of health insurance that they have. The variable health insurance was dichotomized as compulsory to include older adults who only had the universal health insurance, and private insurance to include older adults who had semi-private or private health insurance.

Other type of government support was determined by creating a single variable that combined the affirmative answers to either of these two questions: do you receive supplementary benefits? (yes, no or I don’t know), and/or do you receive helplessness compensation? (yes, no or I don’t know).

Use of formal care was measured by asking the older adult from which kind of care organizations they receive regular support in everyday life. The question of the survey used in this study, that inquired about the organizations that bring support to the older adult in regular basis included the following options of organizations: Spitex (non-profit), Alzheimer’s Association, Parkinson’s association, Diabetes Association, Red Cross Baselland, Pro Senectute. We selected positive answers on the use of the two home care services (Spitex and Pro Senectute) only, as we were interested in organizations that provide home care services while the other organizations are focused in providing support according to the medical condition.

Economic status was determined using the data of the Swiss Federal Tax Administration of 2017. It corresponds to the gross income minus social security contributions (i.e. payments for unemployment insurance and other elements of obligatory social security, also payments to pension funds) and is corrected for household size and composition [22]. The variable was categorized as low income, middle income and high income for the Canton BL, using the tertiles based on the distribution of the sample. The postal code was used to identify the income category for each individual included in the study.

## Predictor variables at the meso level

Use of different support services like meal service, transport services (special transportation for disable people), flat for older adults, nursing care at home, and help with housework was measured by asking older adults about the kind of help that they used or needed in the previous year (2018). This question included a dichotomized answer (yes/no) for each service inquired.

## Predictor variables at micro level

### Enabling factors

The living situation was assessed by asking the older person about the number of family members living with them in the same household. This question was developed by the research team. For the analysis, two categories were created: living alone or living with significant others. Living with significant others included all the older adults who referred to live with the spouse, siblings, adult children, other adults or a professional caregiver.

Socioeconomical status was determined by calculating the individual income of the older adult by dividing the income of the household by the number of people in the household, following the recommendations of the Swiss Centre of Expertise in the Social Sciences (FORS Guide) to measure income in surveys [23]: an equivalence scale is used for the number of people in the household (1.53 for two persons, 1.86 for three persons, and 0.28 for each additional person living in the household) [23]. Categories of the household monthly income were adjusted using the midpoint of the income band (e.g., 4,500 for income category 3,000–6,000) [23]. 2,495 Swiss francs (USD: 2647) was used as a threshold to consider a person at-risk-of-poverty, which represents a disposable income equivalent to less than 60% of the median in Switzerland [24].

The presence of informal care was obtained by asking to the older person their current source of support, with the following options: family members of the same age (e.g. spouse, partner), younger family members (e.g. children, grandchildren), friends and neighbors, or I don’t need. For the analysis, this question was dichotomized (yes/no), where a positive answer grouped the support received by family members of the same or younger age, friends and neighbors.

### Predisposing factors

The socio-demographic characteristics included age, sex and language. Education level was reported according to the Swiss Educational System, but for the analysis of this study was recategorized using the International Standard Classification of Education [25]. According to it, the following categories were created: Less than primary education: No school leaving certificate; Primary education: elementary school; Secondary education: completed training, gymnasium; Tertiary education: university, University of Applied Sciences / Technical University.

Health behaviors were captured through physical activity; alcohol consumption; and smoking status. For physical activity, participants were asked about the frequency of moderate and vigorous physical activity and muscle-strengthening activities practiced in a typical week. The level of physical activity was defined according to the recommendations of the WHO (optimal level: 150 min of moderate-intensity aerobic physical activity per week; or at least 75 min of vigorous-intensity aerobic physical activity per week; or a combination of both; and the practice of exercises to strength the muscles and improve balance more than 3 times per week) [26]. This variable was dichotomized (optimal level/no optimal level) considering as an optimal level an older adult who referred to practice physical activity according to the WHO recommendations. The level of alcohol consumption was captured by inquiring about the amount consumed per day. We considered as chronic high-risk consumption the consumption of 2 standard glasses/day for women or 4 standard glasses for men [27]. For smoking status, older adults were asked about their current smoking status. Three categories were created for the analysis: current consumers, past consumers, or never consumed-. Current consumer included all older adults who smoke daily and not everyday, past consumers included all older adults who smoked in the past but not at the moment and never consumed included all the older adults who referred that they have never smoked.

Health-related measures such as mental health was measured by using the psychological and social domains of the Groningen Frailty Indicator [28]. The psychological domain includes feeling miserable or depressed or feeling a general emptiness, while the social domain includes missing the company of other people and feeling abandoned. These questions included a yes/no answer, that was used for the analysis. Self-perceived health was extracted from the visual analogue scale (EQ VAS) of the EuroQol 5 Dimensions 5 levels (EQ-5D5L) instrument, where scores range from 0 (the worst health you can imagine) to 100 (the best health you can imagine) [29]. Four cut-offs were created (0–25, 26–50, 51–75 and 76–100) following the distribution of the data and according to the guide of the EuroQol, on how to report the EQ VAS score [29]. To identify the level of dependency of the older person we extracted information from the Lawton and Brody scale. This scale determines the level of dependency to perform eight instrumental activities of daily living (mode of transportation, housekeeping, shopping, food preparation, ability to handle finances, responsibility for own medications, ability to use the phone, and laundry) necessary to live independently in the community [30]. The summary score ranges from 0 (low function, dependent) to 8 (high function, independent). Inter-rater reliability was established at 0.85 and criterion validity has been defined using correlations of this scale with four other scales of functional status, identifying significant correlations among them [30].

### Statistical analysis

Descriptive statistics for socio-demographic and health related variables are presented as frequencies, percentages, medians and interquartile ranges [IQR], as appropriate. No means or standard deviations were calculated due to the non-normal distribution of the data. Correlations have been tested using Cramer’s V coefficients to calculate the strength of association between the predictor variables. Bivariate logistic regression analyses were done to explore the individual association of each factor with the outcome variable. Additionally, multiple logistic regression analyses were performed in order to investigate the association of factors at the system (model 1), service (model 2) and individual levels (model 3) with the presence of perceived unmet care needs for home support. Model 4 explored the association of the predictors that were significant in the bivariate and multivariate analysis performed at each level with the outcome variable. For the multiple logistic regression, we used a backward approach, starting with a saturated model (all variables from each level included in the analysis) and gradually eliminating variables until we found a reduced model that best explained the data. The Akaike score (AIC) and the Bayesian Information Criterion (BIC) were used to determine the best model, considering that a lower AIC or BIC score displays a stronger model. The level of significance p-value was set at 0.05. The estimated coefficients of the regression models were transformed to odds ratio (OR). A multilevel analysis was not necessary as we calculated that only 0.30% of the individual variation in the perception of unmet needs for support was due to differences between municipalities (in intraclass correlation -ICC- values less than 5 are indicative of poor correlation).

The percentage of missing values of the outcome variable (perceived unmet needs) was 17%. Additionally, we found an important percentage of missing values in two variables: individual income (21%) and level of dependency (18%). Therefore, under the traditional listwise deletion method we would have had only 79% of the 8,503 older adults in the sample available for analysis. Data was primarily missing due to item nonresponse, and after the analysis of missing patterns, we considered our data to be missing at random (MAR). We identified that older adults with missing values on the outcome variable were those who didn’t have private health insurance or didn’t receive services such as formal care, care at home by a nurse, transportation services or meal services. For the variable individual income, we identified missing values based on age, sex, and education level, while for level of dependency it was age and sex. As our data met the recommendations of Jakobsen and colleagues (2017) for when to use multiple imputation (i.e. missing data is above 5% but below 40%, data was missing not only on the dependant variable, the MCAR assumption could not be plausible, and we consider our data MAR)[31] we applied multiple imputation by chained equations (MICE) to impute missing values. We used the default settings in the mice function to generate 5 imputed datasets.[32]. We used postal code, age, sex, nationality, language, level of education, self-perceived health, type of insurance, and not giving information about income as predictors for the imputation model. We conducted sensitivity analyses to check differences in distribution between the imputed and observed data and ensure that the results were not impacted by the imputation [33]. We are reporting here the data after the imputation. Data analysis was done using R version 4,0.2 [34].

## Results

### Participants characteristics

From the sample of 8,543 individuals, a total of 8,508 older people were included in the study, after excluding 35 cases with answers *I don’t know* for the health insurance and other types of support variables (see additional file 2).

The age of the respondents ranged from 75 to 107 years, with a median age of 81 years; 48.2% were male, and 80.4% had Swiss nationality. More than two-thirds of the individuals had secondary or tertiary education, while the individual income was above the threshold for the majority of them (96.4%). Less than half of the respondents had a private health insurance (45.1%) and only 6.3% needed additional government support. From the respondents, approximately 12% had some level of dependency (a score ≤ 6 in the Lawton Instrumental Activities of Daily Living (IADL) Scale), but only 4.3% mentioned to have unmet needs for support. Support at home for everyday activities, care at home by a nurse and public transportation were the most common professional services used by the older adults; and 50.2% referred to receive informal support from partners, family or friends. Table 1 shows the characteristics of all participants (see Table 1).

**Table 1:** Description of the included older participants (N = 8,508)

Characteristics		n (%)	Median [IQR]
**Economic status by municipality**			
Low income		1941 (22.8%)	
Medium income		4023 (47.3%)	
High income		2544 (29.9%)	
**Health insurance**			
Compulsory		4671 (54.9%)	
Private ^a^		3837 (45.1%)	
**Other type of government support** ^**b**^		532 (6.3%)	
**Use of formal care**		773 (9.1%)	
**Use of professional home care support**			
Nursing care at home		577 (6.8%)	
Help with housework		1299 (15.3%)	
Meal service		217 (2.6%)	
Transport service		433 (5.1%)	
Flat for older adults		80 (0.9%)	
**Age**			81 [78–85]
75–80		4065 (47.8%)	
81–85		2590 (30.4%)	
> 85		1853 (21.8%)	
**Sex**			
Female		4408 (51.8%)	
**Nationality**			
Switzerland		6845 (80.5%)	
Germany		820 (9.6%)	
France		96 (1.1%)	
Other		747 (8.8%)	
**German speaking abilities**			
Swiss German as mother tongue		6636 (78.0%)	
German as mother tongue		1053 (12.4%)	
Good German		718 (8.4%)	
Bad German		101 (1.2%)	
**Education** ^**c**^			
Less than primary education		81 (1.0%)	
Primary education		1253 (14.7%)	
Secondary education		4707 (55.3%)	
Tertiary education		2076 (24.4%)	
Other		391 (4.6%)	
**Unmet needs for home support**		355 (4.2%)	
**Level of physical activity** ^**d**^			
Less than optimal		2946 (34.6%)	
Optimal		5562 (65.4%)	
**Chronic alcohol consumption**			
High-risk		3301 (38.8%)	
Low-risk		5207 (61.2%)	
**Smoking status**			
Never smoked		4746 (55.8%)	
Current consumer		591 (6.9%)	
Past consumer		3171 (37.3%)	
**Mental Health** ^**e**^			
*Psychological domain (GFI)*	Feeling depressed	1298 (15.3%)	
	Feeling anxious	1284(15.1%)	
*Social domain (GFI)*	Feeling empty	2007 (23.6%)	
	Feeling abandoned	890 (10.5%)	
	Missing company of others	3007 (35.3%)	
**Self-perceived health status scores (EQ-5D VAS)** ^**f**^			80 [70–85]
0–25		65 (0.8%)	
26–50		956 (11.2%)	
51–75		2831 (33.3%)	
76–100		4656 (54.7%)	
**Individual income**			
Below threshold ^g^		303 (3.6%)	
**Living situation**			
Living alone		3059 (36.0%)	
**Informal care by family or friends**		4271 (50.2%)	
**Level of dependency (IADL scale)** ^**h**^			8.0 [7.0–8.0]
0–2		78 (0.9%)	
3–4		161 (1.9%)	
5–6		831 (9.8%)	
7–8		7438 (87.4%)	

^a^ Supplementary support and helplessness compensation; ^b^ Semi-private, private and flex health insurance; ^c^ International Standard Classification of Education; ^d^ Optimal physical activity = WHO recommendations; ^e^ GFI = Groningen Frailty Indicator; ^f^ EQ-5D VAS = visual analogue scale of the EQ-5D questionnaire; ^g^ Individual income below threshold = 2,495 Swiss francs (USD: 2647); ^h^ IADL = Lawton Instrumental Activities of Daily Living

### Factors affecting perceived unmet needs for support at home

The bivariate logistic regression showed that having a private health insurance, being male, being proficient in German, having a level of education above primary, keeping a level of physical activity according to the recommendations of the WHO, having a self-perceived health status score above 25, having an income above the threshold, living with a significant other or having an IADL score of ≥ 7 were significantly associated with a lower risk of having perceived unmet needs for home support. On the other hand, receiving other type of support from the government, using nursing care at home and transportation services, or feeling abandoned or depressed were significantly associated with a higher risk for having perceived unmet needs for support at home (see additional file 3).

**Model 1 (AIC = 2898.2)**, which included only the variables at the macro level, showed that having private health insurance was significantly associated with a lower risk for having perceived unmet needs for support at home, while receiving other type of government support significantly increased the risk. **Model 2 (AIC = 2940.9)**, which included only the variables at the meso level, showed that the use of using care at home services and transportation services was significantly associated with having perceived unmet needs for support at home. In **model 3 (AIC = 2807.2)**, where we included the variables at the micro level, it showed that speaking Swiss German or German as a mother tongue, having at least a secondary level of education, keeping an optimal level of physical activity, having chronic-high risk alcohol consumption, having a score of self-perceived health status above 51, having an income above the threshold and having informal care lowered the risk of having perceived unmet needs for support at home. On the other hand, feeling depressed and feeling abandoned were significantly associated with having perceived unmet needs for support at home (see additional file 3).

Table 2 presents the odds ratio of the **Model 4 (AIC = 2773.3**), which included all the significant variables of the bivariate and models 1, 2 and 3. This model showed that having private health insurance, speaking Swiss-German or German as a mother tongue, having a level of education above primary, keeping an optimal level of physical activity according to the recommendations of the WHO, having a chronic-high risk alcohol consumption, having a score of self-perceived health status above 76 and having informal care was associated with a lower risk of having perceived unmet needs for support at home. On the contrary, receiving other type of government support, using transportation services, feeling depressed and feeling abandoned were significantly associated with having perceived unmet needs for support at home (see Table 2).

**Table 2:** Multiple logistic regression of perceived unmet needs for support at home by level (macro, meso, and micro) model 4 (N = 8,508)

		Model 4 OR (95% CI) AIC = 2773.3 BIC = 2928.3
**Macro level**		
**Economic status by municipality (ref. low income)**		
Middle-income		
High-income		
**Private Health insurance (ref. Compulsory insurance)**		0.63 (0.49–0.80) *
**Other type of government support (ref. no support)**		1.65 (1.17–2.29) *
**Use of formal care (ref. no)**		
**Meso level**		
**Services used (ref. no)**		
Nursing care at home		
Meal service		
Transport service		1.74 (1.15–2.57) *
Flat for older adults		
Help with housework		
**Micro level**		
**Male (ref. female)**		0.85 (0.65–1.11)
**Age (ref. 75–80)**		
81–85		1.04 (0.81–1.34)
> 86		0.80 (0.58–1.08)
**German speaking proficiency (ref. bad German)**		
Swiss German as mother tongue		0.44 (0.24–0.88) *
German as mother tongue		0.47 (0.24–0.98) *
Good German		0.64 (0.32–1.34)
**Education (ref. less than primary)**		
Primary education		0.48 (0.24–1.02) *
Secondary education		0.49 (0.25–1.03) *
Tertiary education		0.38 (0.19–0.82) *
Other		0.31 (0.12–0.75) *
**Physical activity (ref. no optimal)**		
Optimal		0.77 (0.61–0.96) *
**Chronic alcohol consumption (ref. low risk)**		
High-risk		0.76 (0.58–0.99) *
**Smoking status (ref. never smoked)**		
Current consumer		
Past consumer		
**Mental Health (ref. no)**		
*Psychological domain*	Feeling depressed	1.40 (1.06–1.85) *
	Feeling abandoned	2.60 (1.96–3.43) *
**Self-perceived health status (ref. 0–25)**		
26–50		0.60 (0.29–1.38)
51–75		0.50 (0.24–1.12)
76–100		0.42 (0.20–0.96) *
**Individual income (ref. below threshold)**		
Above threshold		
**Living situation (ref. living alone)**		
Living with a significant other		
**Informal care (ref. no informal care)**		0.57 (0.45–0.73) *
**Level of dependency (ref. ≤ 2)**		
3–4		
5–6		
7–8		

*P < 0.05

## Discussion

Using data of the INSPIRE Population Survey, we conducted a secondary analysis to assess the multi-level factors associated with unmet needs for home support among 8,508 home-dwelling older adults. We found that only 4.2% of older adults in the canton Basel-Landschaft perceived having unmet needs for home support, which is low compared to the prevalence reported in other high-income countries. For example, Canada reported a prevalence of perceived unmet needs for home support among older adults of 75 to 79 years of approximately 58%; however they considered the presence of unmet needs for home support when the person or anyone in their household felt that home support was needed but was not received [4]. The UK, on the other hand, which determines the presence of unmet needs of home support based on the reported difficulty to perform one or more ADL or IADL, has reported a presence of unmet needs in older adults that ranges from 12.5% in the presence of difficulty to perform more than three ADL to 55% when only one ADL is considered [3]. The differences in the prevalence of unmet needs for home support in these high-income countries could be explained by the absence of a standardized definition of unmet needs for home support that can lead to an imprecise assessment and an overestimation of the real problem, as none of these measurements incorporates the perspective of the older adult about their needs [3, 6]. This highlights the importance of combining objective measurements and older adults’ perspectives in longitudinal studies to obtain a more precise determination of the prevalence, considering that the onset of needs for home support in home-dwelling older adults can vary over time.

The multiple regression analysis, following an ecological approach, demonstrated that macro and meso level factors are correlated with unmet needs for home support and confirmed existing evidence about the role of micro level factors on the perception of unmet needs for home support.

We could identify that at the macro level, older adults who hold a private health insurance were less likely to have perceived unmet needs for home support than those with only a compulsory health insurance. Similar findings have been reported in a study among adults from 18 to 65 + years in the US, where higher levels of unmet needs for home support were likely to be present in uninsured individuals [35]. Traditionally, private health insurances can help to cover services not, or not entirely, covered by compulsory health insurances [36], and access to them is highly determined by the income level [36–38]. In our study only 45% of older adults had a private health insurance, which could somehow reflect the presence of economic inequality. Previous studies have shown the impact of economic inequality in the access to care, where people with lower incomes are significantly less likely to look for health and care services needed compared to those with higher incomes [39, 40]. In Switzerland, approximately 24% of older adults only receive the income provided by the first pillar of the old-age pension system (including other types of government support), being at risk of poverty, with less money to consume and pay, for example, for a private health insurance [15]. This could also explain why even when the older adults received other types of government support, they were more likely to have a higher perception of unmet needs for home support. It is noteworthy that we found these associations between individual unmet needs for home support and factors at the macro level in a high-income country like Switzerland. We therefore hypothesize that these determinants will have a stronger impact on individual unmet needs for home support in older adults residing in low- and middle-income countries.

Our analysis revealed that at the meso level, only the use of transportation services (e.g. taxi, or door-to-door service for people with physical disabilities) was significantly associated to a higher perception of unmet needs for home support. The capacity to move from one place to another is crucial in old age, as it allows an older adult to meet basic needs, access healthcare services and maintain social interactions [41, 42]. A higher use of transportation services is often reported among older adults due to environmental characteristics, mobility limitations or the lack of a driver in their support network [42, 43]. Basel-Landschaft is traditionally considered an urban/rural canton, with greater geographical distances to access services and limited public transportation, making the use of transportation services more likely. The positive association that we found between unmet needs for home support and the use of transportation services could be related to the costs to access transport service. In Switzerland, the use of transportation services is subject to a direct payment, as it is not covered by the compulsory health insurance [44]. There could be circumstances when an older adult would prefer to pay for transportation services instead of other services, with the risk of developing unmet needs for home support. However, as we didn’t have additional information regarding the frequency of the use of the service, these results need to be interpreted with caution.

At the micro level, we found that speaking Swiss German or German, having an education level above primary, having a chronic alcohol consumption, practicing an optimal level of physical activity, having a self-perceived health status above 76, and relying on an informal caregiver decreased the risk of perceiving unmet needs for home support in home-dwelling older adults. In the other hand, mental health factors such as feeling depressed or abandoned, increased the risk of perceiving unmet needs for home support. Our findings about language proficiency and education level are in line with previous studies that have shown how social disparities can place vulnerable people in a more disadvantageous situation [45]. For example, studies in Canada, UK, Norway and the U.S. have shown that language and education can become a barrier to navigate in the care system, either because older adults are not aware of the services, or due to the difficulty to communicate with others [3, 6, 11, 46]. Another explanation for these findings may be associated to the fact that a good level of education allows a person to contribute a higher amount to the pension system and keep private retirement savings [47, 48], so their incomes in old age are enough to cover their needs for support [15].

Although in our study the association between the living situation and the presence of unmet needs for home support was not significant, other studies have determined that it can be a strong predictor of unmet needs [3, 4]. Living alone can also lead to the development of depression, feelings of abandonment, and the perception of unmet needs, as older adults do not have the amount of social interaction that they would like to have [9]. This coincides with the results of our multivariate analysis where older adults who were feeling depressed and/or abandoned were at a higher risk of having perceived unmet needs for home support, while those who rely on an informal caregiver showed a lower perception of unmet needs for support. Two hypotheses could explain this association between informal care and unmet needs for home support. First, older adults who are recipients of informal care are less likely to fully acknowledge or admit their needs for care and support, and take pride of being independent and able to take care of themselves [9]. Second, older adults living with the caregiver show a lower perception of unmet needs, as they can receive the support for little things that make their life more enjoyable, such as reminding them of medication, helping with eye drops, going with them to medical appointments, etc., whenever such support is needed. [9]. However, in our study we did not have further information about the informal caregivers, thus this deserves more attention in future studies.

In accordance to previous studies, our analysis demonstrated that a good self-rated health status and health behaviours such as an optimal level of physical activity were associated with a lower perception of unmet needs for support at home [2, 11, 12]. Although the use of questionnaires to measure physical activity in older adults is a widespread practice in large studies, to date there is no questionnaire with sufficient content validity, and reliability [49]. Thus, we could assume that some older adults could have over- or underestimated their physical activity levels. We also found a negative association between a problematic alcohol consumption and the perception of unmet needs for support at home. A possible explanation could come from the tendency to drink more observed in those older adults with a good perception of their own health, while those with a poor self-rated health status avoid the consumption of alcohol [50, 51]. Another explanation could be related to the fact that alcohol consumption is seen as a mean for enhancing social engagement with friends and relatives [52].

### Methodological considerations

The strength of this study was the use of a population-based dataset to identify the prevalence and factors associated with perceived unmet needs for home support in older adults of a developed country using an ecological approach. As such, having a large sample size of 8,508 individuals allowed us to increase the accuracy of the estimates of the regression analysis. Yet, findings need to be seen in light of certain limitations. First, the major limitation of this study is the use of secondary data for the analysis. Second, the response rate of the original survey could be considered as low and hence a source of bias. However, this response rate was much higher than those previously reported using postal mail as a delivery method in population surveys without any direct incentive for participation [53]. Additionally, we recognize that due to anonymous data collection used in this survey, we had no means of knowing whether the non-respondents were frail or cognitively impaired, which may affect the response rate, thus subjecting this study to additional selection bias. Third, the data used to determine the prevalence of perceived unmet needs for home support might not be enough to estimate the real magnitude of unmet needs among the Swiss older population. First, as the original data come from a cross-sectional study, we might be reporting the patterns of unmet needs for home support under a specific time and circumstances. The onset of needs is an unsteady process, as they can appear as a sudden incident, be the result of a chronic condition, age-related physiological changes or due to the loss of a spouse or source of support [9]; thus, a cross-sectional approach might not be adequate for determining the prevalence of unmet needs for home support. Also, we used a single self-reported dichotomous variable to compute the presence of perceived unmet needs for home support: the support provided matches their everyday life needs. We did not have additional information about the type of needs (e.g., health vs. social), how often these needs were not met or their reasons why these needs were not met. Thus, we could be underestimating the presence of perceived unmet needs. Therefore, future studies should focus on using a definition of unmet needs that combines an objective measurement with the perspective of the older adult to understand the dynamics behind the perception of unmet needs for support among older adults in longitudinal studies. This new approach could provide us with a deep understanding of the reasons for unmet needs. Another consequence of the use of secondary data was the limited information available to determine the factors of the macro and meso level that were associated with the perception of unmet needs, using an ecological approach. Although we incorporated the average income per municipality as a way to enhance the macro level, and the use of different formal care services at the meso level this could have been not enough to represent the socio-economical situation and the dynamics behind the provision of formal care services in BL. Therefore, future studies could incorporate more socio-economical factors at the macro level and precise information about the provision of formal care at the meso level as our study demonstrated that factors at both levels are associated with the perception of unmet needs. Finally, given the federal system in Switzerland, where services are also organized at canton and municipality level, the generalizability of the results might be limited.

## Conclusion

This study set out to determine the prevalence and the multi-level factors associated with the presence of perceived unmet needs for home support. Our findings determined a low prevalence of perceived unmet needs in home-dwelling older adults of a high-income country like Switzerland, despite the methodological considerations for its measurement mentioned above. Additionally, the findings in this study highlight the role of socio-economical inequalities at different levels in the perception of unmet needs for home support among home-dwelling older adults, validating the use of ecological approaches for the analysis.

Overall, these findings provide valuable information for policy makers. Our findings show that socio-economical inequalities seem to play an important role in the perception of unmet needs for home support; hence a number of specific policies and practices should be addressed to reduce inequalities. For example, fiscal and monetary policies, or strategies to reduce out of pocket-payments should be considered. Providing higher incomes to home-dwelling older adults could allow them to pay for additional health and social care services not currently covered, and therefore reduce their perception of unmet needs.

Our findings also highlight the importance of obtaining the perspective of the older adult in the assessment of their needs for home support, and developing targeted interventions to improve the access to different care services and financial benefits in the community. This information supports the implementation of community-based integrated care models, like the INSPIRE care model, which is being implemented in the Canton BL.

## Electronic supplementary material

Below is the link to the electronic supplementary material.

Supplementary Material 1
Additional fie 1. Table 1. Levels, categories and variables used for the analysis.
Additional fie 2. Flowchart of sample selection.
Additional file 3. Table 2. Bivariate logistic regression and multiple logistic regression (models 1-3) of perceived unmet needs for home support by level (macro, meso, micro) (N=8,508).

## Data Availability

The dataset used in the current study (INSPIRE Population Survey) is not publicly available due to it corresponds to a vulnerable population but can be requested to the corresponding author on reasonable request. The economics status per municipality data (Municipality’s average equivalent taxable income), can be accessed in the official site of the Swiss Federal Tax Administration (https://www.estv.admin.ch/estv/de/home.html).

## Declarations

As this is a secondary analysis of the INSPIRE Population survey, the approval of the ethics committee for the present study was not required.

## Abbreviations

INSPIRE: ImplemeNtation of a community-baSed care Program for home dwelling senIoR citizEns.
Canton BL: Canton Basel-Landschaft.
ADL: Activities of daily living.
IADL: Instrumental activities of daily living.
ICOPE: Integrated Care for Older People.
