# Supplementary material for: A multi-level perspective on perceived unmet needs for home support in home-dwelling older adults in the Swiss context: a secondary data analysis of a population study
Source: BMC Geriatr. 2022 Nov 3;22:833. doi: 10.1186/s12877-022-03479-5 (PMC9635119; doi:10.1186/s12877-022-03479-5)
Supplement: Supplementary file 1 — Supplementary Material 1 Additional fie 1. Table 1. Levels, categories and variables used for the analysis. Additional fie 2. Flowchart of sample selection. Additional file 3. Table 2. Bivariate logistic regression and multiple logistic regression (models 1-3) of perceived unmet needs for home support by level (macro, meso, micro) (N=8,508). [file 12877_2022_3479_MOESM1_ESM.docx]

**Supplementary information**

**Additional file 1**

**Table 1.** Levels, categories and variables used for the analysis.

| **Domain** | **Variable** | **Scale and reference (if applicable)** | **Items** | **Answer options** | **Psychometrics (if available)** |
| --- | --- | --- | --- | --- | --- |
| **Outcome variable** | | | | | |
| Unmet needs | Support received |  | Support received in everyday life | •       Yes, I am getting the support I need   - No, I need more support |  |
| **Macro level** | | | | | |
| Health insurance | Health insurance |  | Type of health insurance that the older adult has | - Compulsory - Private - No insurance |  |
| Other type of government support | Other type of government support |  | Recipient of supplementary benefits and/or helplessness compensation | - Yes / No /Don't know |  |
| Use of formal care | Provision of formal care |  | Use of formal care services (i.e. Spitex and Pro Senectute) | - Yes / No |  |
| Economic status by municipality | Municipality’s average equivalent taxable income | Swiss Federal Tax Administration, 2017 [22] | Gross income minus social security contributions (i.e. payments for unemployment insurance and other elements of obligatory social security, also payments to pension funds) and is corrected for household size and composition | - Low income - Middle income - High income |  |
| **Meso level** | | | | | |
| Use of support services | Support services |  | Support received in the previous year by different providers | - I didn't need any help in 2018 - Nursing care at home (Public Organization or Private Organization) - Help with the housework (Public Organization or Private Organization) - Meal service - Transport and assistance |  |
| **Micro level** | | | | | |
| *Predisposing factors* | | | | | |
| Socio-demographics | Age |  | Year of birth |  |  |
|  | Sex |  | Sex | - Male - Female |  |
|  | Education level |  | Highest level of education | - No school-leaving qualification - Elementary school - Completed education - High school - Technical college / technical university - University - Don't know - Other |  |
|  | Nationality |  | Country of birth | - Switzerland - France - Germany - Others |  |
| Health behaviors | Level of physical activity |  | Moderate and vigorous practice of physical activity and muscle-strengthening activities practice in a typical week | Moderate/vigorous physical activity   - Less than 30 minutes - 30-74minutes - 75 minutes or more   Muscle-strengthening   - At least once a week - Less than once a week - Never |  |
|  | Chronic alcohol consumption | Chronic high-risk consumption is the consumption of 2 standard glasses/day for women or 4 standard glasses per day for men [27]. | Amount of alcoholic consumption per day | - 0 beverages - 1-2 drinks - 3-4 drinks - 5 or more drinks |  |
|  | Smoking status |  | Current or past consumption | - Yes, daily - Yes, not everyday - No, but I was a smoker - No, never |  |
| Mental health | Phycological domain | Groningen Frailty Indicator [23] | - Feeling miserable or depressed - Feeling a general emptiness | - Yes / No | Pearson r = 0.556 [49] |
|  | Social domain |  | - Missing the company of other people - Feeling abandoned | - Yes / No | Pearson r = 0.534 [49] |
| Health status perception | Self-perceived health status | EQ 5D 5L Scores range from 0 (the worst health you can imagine) to 100 (the best health you can imagine) [24]. | Current health perception | - Scale from 0 to 100 | test–retest reliability s = 0.78 [38] |
| Needs | Level of dependency | Lawton-Brody scale [27]. The summary score ranges from 0 (low function, dependent) to 8 (high function, independent) | Mode of transportation | - I ride a bicycle / e-bike I drive my own car - I use public transport - I order and use a taxi on my own, but no public transport - I use public transport in company - I make limited journeys in a taxi or car in company - I can't move outside the house anymore | Inter-rater reliability = 0.85, |
|  |  |  | Housekeeping | - I don't have any problems with my daily activities. - I have slight problems with my daily activities - I have moderate problems with my daily activities. - I have big problems to do my everyday activities - I am not in a position to pursue my everyday activities |  |
|  |  |  | Shopping | - I can do all my shopping independently - I can only do small purchases independently - I need help shopping - I'm not able to do any shopping |  |
|  |  |  | Food preparation | - I plan and cook meals independently - I need help preparing meals - I warm up the meals prepared by other people - The meals must be prepared ready to eat |  |
|  |  |  | Ability to use the phone | - I use the phone independently - I'm just dialing some known numbers - I pick up the phone, but I don't dial on my own - I don't use the phone at all |  |
|  |  |  | Do the laundry | - I can wash the laundry myself - I can do small laundry, e.g. socks wash - My laundry must be done completely by others |  |
|  |  |  | Ability to handle medications | - I am taking my medication on my own in exact dosage and at the correct time - I take prepared medications correctly (e.g. in doses) - I cannot manage the correct intake of medication on my own. |  |
|  |  |  | Ability to handle finances | - I manage financial transactions independently (budget, cheques, deposit, bank transfer) - I can make the daily, smaller expenses, but I need help with transfers and bank transactions - I'm no longer able to handle money |  |
| *Enabling factors* | | | | | |
| Socioeconomical status | Individual income | 2.495 Swiss francs was used as a threshold to consider a person at-risk-of-poverty. It represents a disposable income equivalent to less than 60% of the median in Switzerland [24] | Monthly income of the older person | - Below threshold - Above threshold |  |
| Living situation | Living arrangements |  | Number of people living in the same household with the older person | - Number of people living in the same house |  |
| Informal caregiver | Source of care |  | Source of regular support in everyday life | - Family members of the same age (e.g. spouse, partner) - Younger family members (e.g. children, grandchildren) - Friends and neighbors - I don't need |  |

**Additional fie 2**

**Flowchart of sample selection**

**
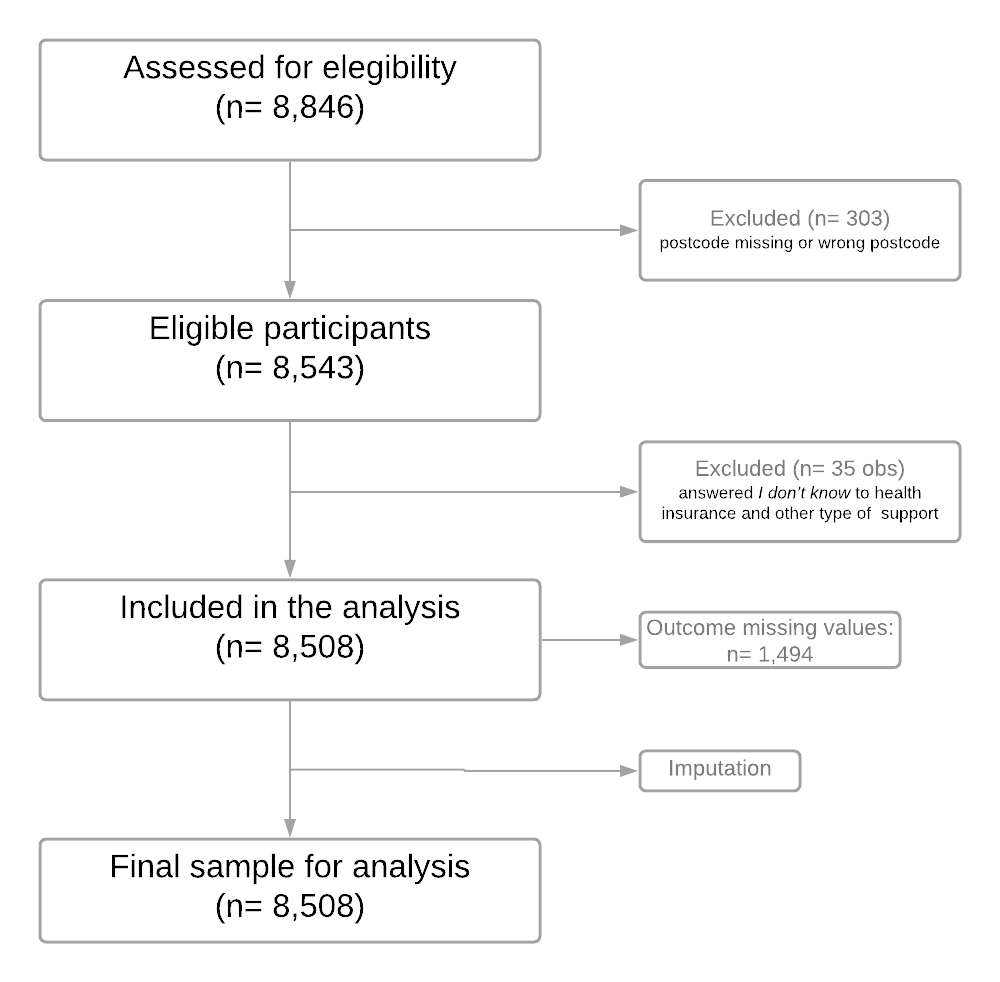
**

**Additional file 3**

Table 2. Bivariate logistic regression and multiple logistic regression (models 1 -3) of perceived unmet needs for home support by level (macro, meso, micro) (N=8,508)

|  | | **Bivariate analysis**  **OR (95% CI)** | **Model 1**  **OR (95% CI)**  **AIC= 2898.2**  **BIC=** **2940.5** | **Model 2**  **OR (95% CI)**  **AIC= 2940.9**  **BIC= 2983.2** | **Model 3**  **OR (95% CI)**  **AIC=** **2807.2**  **BIC= 2990.5** |
| --- | --- | --- | --- | --- | --- |
| **Macro level** | |  |  |  |  |
| **Income by municipality (ref. low income)** | |  |  |  |  |
| Middle-income | | 1.11 (0.85 - 1.46) | 1.22 (0.93 - 1.60) |  |  |
| High-income | | 0.87 (0.64 - 1.19) | 1.07 (0.78 - 1.46) |  |  |
| **Private Health insurance (ref. Compulsory insurance)** | | 0.52 (0.41 – 0.65) * | 0.55 (0.43 - 0.70) * |  |  |
| **Other type of government support (ref. no support)** | | 2.81 (2.05 – 3.79) * | 2.44 (1.76 - 3.32) * |  |  |
| **Use of formal care (ref. no)** | | 1.36 (0.97-1.87) | 1.12 (0.79 – 1.56) |  |  |
| **Meso level** | |  |  |  |  |
| **Services used (ref. no)** | |  |  |  |  |
| Nursing care at home | | 1.69 (1.18 – 2.37) * |  | 1.49 (0.99 – 2.21) * |  |
| Meal service | | 0.88 (0.39 – 1.67) |  | 0.59 (0.26 – 1.18) |  |
| Transport service | | 2.20 (1.52 – 3.12) * |  | 2.06 (1.37 – 3.01) * |  |
| Flat for older adults | | 0.59 (0.10 – 1.87) |  | 0.57 (0.09 – 1.83) |  |
| Help with housework | | 1.21 (0.91 – 1.58) |  | 0.99 (0.71 – 1.34) |  |
| **Micro level** | |  |  |  |  |
| **Male (ref. female)** | | 0.75 (0.60 – 0.93) * |  |  | 0.83 (0.62 – 1.10) |
| **Age (ref. 75-80)** | |  |  |  |  |
| 81-85 | | 1.19 (0.94 – 1.51) |  |  | 1.05 (0.81 - 1.34) |
| >86 | | 0.93 (0.69– 1.23) |  |  | 0.78 (0.56 - 1.07) |
| **German speaking proficiency (ref. bad German)** | |  |  |  |  |
| Swiss German as mother tongue | | 0.21 (0.12 - 0.37) * |  |  | 0.44 (0.23 - 0.88) * |
| German as mother tongue | | 0.26 (0.14 - 0.49) * |  |  | 0.48 (0.24 – 0.99) * |
| Good German | | 0.31 (0.17 - 0.60) * |  |  | 0.62 (0.31 – 1.30) |
| **Education (ref. less than primary)** | |  |  |  |  |
| Primary education | | 0.27 (0.15 - 0.52) * |  |  | 0.51 (0.25 - 1.08) |
| Secondary education | | 0.21 (0.12 - 0.40) * |  |  | 0.47 (0.24 - 0.99) * |
| Tertiary education | | 0.14 (0.08 - 0.28) * |  |  | 0.33 (0.16 - 0.71) * |
| Other | | 0.15 (0.07 - 0.34) * |  |  | 0.30 (0.12 - 0.73) * |
| **Physical activity (ref. no optimal)** | |  |  |  |  |
| Optimal | | 0.63 (0.50 - 0.77) * |  |  | 0.75 (0.60 - 0.94) * |
| **Alcohol consumption (ref. no problematic)** | |  |  |  |  |
| Problematic | | 0.81 (0.64 – 1.01) |  |  | 0.72 (0.55 - 0.93) * |
| **Smoking status (ref. never smoked)** | |  |  |  |  |
| Consumer | | 0.91 (0.57 - 1.38) |  |  | 0.91 (0.57 - 1.41) |
| Past consumer | | 0.95 (0.76 - 1.19) |  |  | 1.01 (0.79 - 1.29) |
| **Mental Health (ref. no)** | |  |  |  |  |
| *Psychological domain* | Feeling depressed | 2.49 (1.96 - 3.14) * |  |  | 1.45 (1.09 - 1.91) * |
|  | Feeling abandoned | 3.88 (3.04 - 4.92) * |  |  | 2.72 (2.05 - 3.58) * |
| **Self-perceived health status (ref. 0-25)** | |  |  |  |  |
| 26-50 | | 0.40 (0.20 – 0.87) * |  |  | 0.57 (0.27 – 1.32) |
| 51-75 | | 0.26 (0.13 – 0.54) * |  |  | 0.45 (0.21 – 1.04) * |
| 76-100 | | 0.19 (0.10 – 0.40) * |  |  | 0.38 (0.18 - 0.88) * |
| **Individual income (ref. below threshold)** | |  |  |  |  |
| Above threshold | | 0.47 (0.31 – 0.73) * |  |  | 0.62 (0.40 - 1.00) * |
| **Living situation (ref. living alone)** | |  |  |  |  |
| Living together | | 0.78 (0.63 - 0.97) * |  |  | 0.92 (0.72 - 1.18) |
| **Informal care (ref. no informal care)** | | 0.87 (0.70 - 1.07) |  |  | 0.59 (0.46 - 0.76) * |
| **Level of dependency (ref. ≤ 2)** | |  |  |  |  |
| 3-4 | | 0.97 (0.36 – 2.87) |  |  | 1.60 (0.55 – 5.14) |
| 5-6 | | 0.69 (0.30 - 1.84) |  |  | 1.45 (0.58 – 4.26) |
| 7-8 | | 0.49 (0.23 – 1.27) * |  |  | 1.15 (0.47 – 3.35) |

**P* < 0.05
